# The Effect of Captopril on Impaired Wound Healing in Experimental Diabetes

**DOI:** 10.1155/2012/785247

**Published:** 2012-07-24

**Authors:** Ehsan Zandifar, Sajedeh Sohrabi Beheshti, Alireza Zandifar, Shaghayegh Haghjooy Javanmard

**Affiliations:** ^1^Physiology Research Center, Department of Physiology, Isfahan University of Medical Sciences, Isfahan, Iran; ^2^Isfahan Medical Student Research Center, Isfahan University of Medical Sciences, Isfahan, Iran; ^3^Department of Physiology, Isfahan Payame Noor University, Isfahan, Iran

## Abstract

We aimed to investigate whether oral administration of captopril modulate wound healing, nitric oxide (NO), and vascular endothelial growth factor (VEGF) concentration in wound fluid of diabetic rats. 48 male Sprague-Dawley rats were divided in four groups (*n* = 12). The 36 rats were rendered diabetic by streptozotocin. The animals of the first and second groups received 25 and 50 mg/kg/day captopril, respectively, (DM-cap25 and DM-cap50). The animals of the third group were treated by distilled water (DM-control). Control rats had no intervention. The wound fluid level of NO and VEGF were measured. Wound specimens were investigated histopathologically. At the 5th day, there was significantly more NO_*x*_ in wound fluid of DM-cap25 compared to other groups. At the 7th day, both captopril-treated groups had more NO_*x*_ in wound fluid compared to other groups. At the 11th day, both captopril-treated groups had more NO_*x*_ in wound fluid compared to DM-control group. VEGF concentration was significantly higher in both captopril-treated groups versus DM-control group (*P* < .05). There were significant higher wound healing scores in captopril-treated groups compared with DM-control group (*P* < .05). These results suggest that captopril might be useful in diabetic wound healing.

## 1. Introduction

Impaired wound healing is a substantial problem in both type 1 and type 2 diabetes. The wound is often resistant to conventional wound management and may ultimately threaten limb viability [1]. Analysis of the diabetic wound microenvironment has revealed that healing impairment is characterized by a number of local cytokine and cellular abnormalities such as reduced angiogenesis, decreased collagen synthesis and wound breaking strengths [2]. Growth factors such as platelet-derived growth factor (PDGF) and vascular endothelial growth factor (VEGF) have been found to be diminished in the diabetic wound, whereas levels of matrix metalloproteinase and superoxide are elevated in diabetic wound fluid [3, 4]. 

The diabetic wound also demonstrates a number of abnormalities reminiscent of endothelial dysfunction. For example, reduced wound nitric oxide (NO) concentration has been shown in experimental diabetic animal models of acute cutaneous wound healing [5]. Diabetic wounds have also evident reduction in cutaneous blood flow, and abnormal angiogenesis supports the theory of impaired endothelial function and consequently delayed wound repair [6, 7]. So, restoration of endothelial function may be a good therapeutic approach in the diabetic wound healing process.

Angiotensin-converting enzyme inhibitors (ACEIs) have known restorative effects on endothelial cell functions. ACEIs enhanced endothelial cell survival through activation of prosurvival signals such as Akt phosphorylation and endothelial NO synthase (eNOS) expression. However, ACEIs block the production of angiotensin II (Ang II) which has many proangiogenic activities [8, 9]. ACEIs are recommended for the prevention of chronic kidney disease in diabetic patients, while the effect of ACEIs on diabetic wound healing is still unclear.

A few studies have investigated the potential safety concerns with respect to angiogenesis. We aimed to investigate whether oral administration of captopril modulate wound healing and NO and VEGF concentration in wound fluid in an acute incisional wound model in diabetic rats.

## 2. Methods

### 2.1. Animals and Experimental Protocol

48 male Sprague-Dawley rats, weighing 180 to 220 g, from Razi Institute of Iran, were housed one per cage, maintained under controlled environmental conditions (12-hour light/dark cycle, temperature approximately 23°C), and provided with standard laboratory food and water ad libitum. The study protocol was approved by Institutional Animal Ethics Committee according to principles of laboratory animal care [10].

Diabetes was induced by a single 65 mg/kg intravenous injection of streptozotocin (STZ, Sigma, USA) in saline-sodium citrate buffer (Sigma, Inc., St. Louis, MO, USA, pH 4.5) to 36 rats. Blood glucose levels were measured using a glucometer (GR-102, TERUMO Co., Tokyo, Japan). Two weeks after STZ injection, animals with blood glucose levels above 300 mg/dL were defined as diabetic and used in the study. The animals were divided into four groups: three diabetic and one nondiabetic. Each group had 12 animals. The animals of the first diabetic group received captopril (25 mg/kg/day) (DM-cap25). The animals of the second diabetic group received captopril (50 mg/kg/day) (DM-cap50), while the animals of the third diabetic group received only distilled water (DM-control) for 11 days through gavage. The fourth group, consisting of nondiabetic rats, were given distilled water, by gavage for 11 days (non-DM-control). The doses were selected based on work by other studies [11, 12].

In the first day of the study, after general anesthesia with sodium pentobarbital (80 mg/kg, ip), hair on the back was shaved and the skin was washed with a povidone-iodine solution and then wiped with sterile water. Full-thickness wounds (1 cm^2^) were made on the dorsum of the rats.

By the end of the study, the fasting plasma glucose (FPG) was measured again in all animals by enzymatic method (Boehringer Mannheim, Germany).

### 2.2. Determination of VEGF and NO in Wounds

Two samples of wound fluid were collected using sterile nitrate-free absorbent paper strips placed on the edges of the wound for 10 min, in order to measure VEGF in the 5th day and NO levels in 5th, 7th, and 11th days of the study. This method for the measurement has been validated for other sample types, particularly for tears [13, 14]. For VEGF measurement, protein elution from the Shirmer strips was performed by stirring the strips in 0.5 mL of buffer (50 mM Tris, 50 mM NaCl, 0.05% Brij 35, pH 7.6) for at least 2 h at +4°C. For wound fluid NO_*x*_ determinations, filter paper was placed in 0.5 mL of distilled water [15].

The amount of VEGF in wound fluid was measured using enzyme-linked immunosorbent assay using available reagents and recombinant standards (R&D Systems, Minneapolis, MN, USA) according to manufactures instruction only in 5th day samples. The VEGF assay has a minimum sensitivity of 3.0 pg/mL. The total nitrite level of wound fluid was measured using the Griess assay after conversion of NO_3_ to NO_2_ with the NO_3_ reductase enzyme as described previously [16].

### 2.3. Histologic Examination

All wound tissue specimens were fixed in 10% neutral-buffered formalin for at least 24 h at room temperature. After fixation, vertical sections to the anterior-posterior axis of the wound were dehydrated in graded ethanol, cleared in xylene, and embedded in paraffin. Four-micron-thick sections were mounted on glass slides, dewaxed, rehydrated to distilled water, and stained with hematoxylin and eosin. For histological evaluation, all slides were examined by two pathologists, without knowledge of the prior treatment, under a microscope from  ×20  to  ×100 magnification. The histological score adopted in this study was performed according to the previous study concerning wound healing in experimental models. The criteria used as histological scores of wound healing are summarized in Table 1 [17].

### 2.4. Statistical Analysis

All data are expressed as the mean ± the standard deviation (mean ± SD). A statistical software package, SPSS (version 14), was used to perform statistical analysis. The data were tested for normality and homogeneity of variance. Data were analyzed by analysis of variance (ANOVA), followed by a post hoc multiple comparison. For the histological results, statistical analysis was performed using kruskal-Wallis test. Statistical significance was accepted at *P* < 0.05.

## 3. Results

The mean of FPG levels has been illustrated in Table 2. By the end of the study, there was no significant difference between diabetic groups, while all of the diabetic groups had significantly more FPG than non-DM control group (*P* < 0.001).

**Figure 1:**
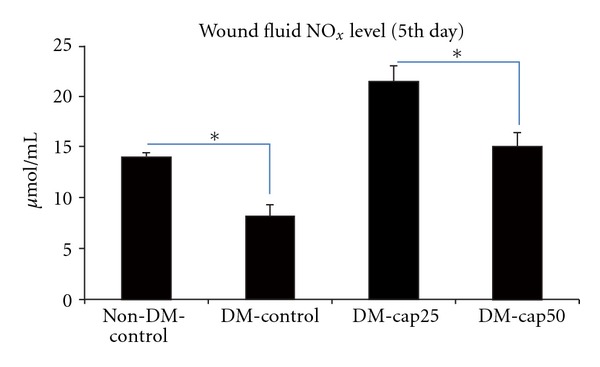
The total nitrite level of wound fluid was measured using Griess assay at the 5th day of study. Non-DM-control: nondiabetic rats which have received distilled water. DM-control diabetic rats which have received distilled water. DM-cap25: diabetic rats which have received captopril (25 mg/kg/day). DM-cap50: diabetic rats which have received captopril (50 mg/kg/day). Data are reported in  *μ*mol/L ± SD (*P < 0.05).

Total nitrite/nitrate concentrations (NO_*x*_) in biological fluids can be used as an index of NOS activity [18]. As it has been illustrated in Figure 1, at the 5th day DM-cap25 group had significantly more wound fluid NO_*x*_ level than other groups (*P* < 0.001). DM-cap50 group had significantly more wound fluid NO_*x*_ level than DM-control group (*P* < 0.001), though there was no significant difference between DM-cap50 group compared to non-DM-control group. The NO_*x*_ level of wound fluid in non-DM-control group was significantly more than DM-control group (*P* < 0.001).

**Figure 2:**
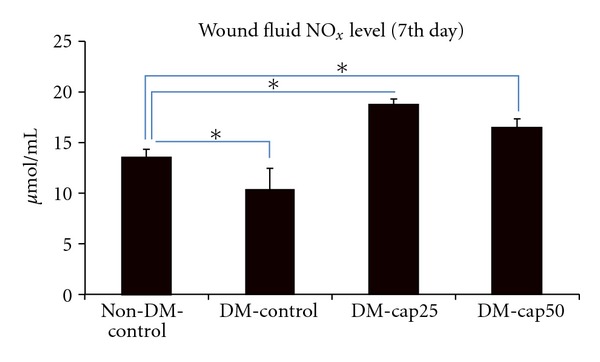
The total nitrite level of wound fluids was measured using Griess assay at the 7th day of study. Non-DM-control: nondiabetic rats which have received distilled water. DM-control: diabetic rats which have received distilled water. DM-cap25: diabetic rats which have received captopril (25 mg/kg/day). DM-cap50: diabetic rats which have received captopril (50 mg/kg/day). Data are reported in  *μ*mol/L ± SD (*P < 0.05).

 At the 7th day, the DM-cap25 group had significantly more wound fluid NO_*x*_ level than DM-control (*P* < 0.001) and non-DM-control groups (*P* < 0.003), while the difference of wound fluid NO_*x*_ level between DM-cap25 group and DM-cap50 group was not significant. The NO_*x*_ level of wond fluid in DM-cap50 group was significantly more than DM-control group (*P* < 0.001) and non-DM-control group (*P* < 0.05). Non-DM-control group had significantly more wound fluid NO_*x*_ level than DM-control group (*P* < 0.05) (Figure 2).

**Figure 3:**
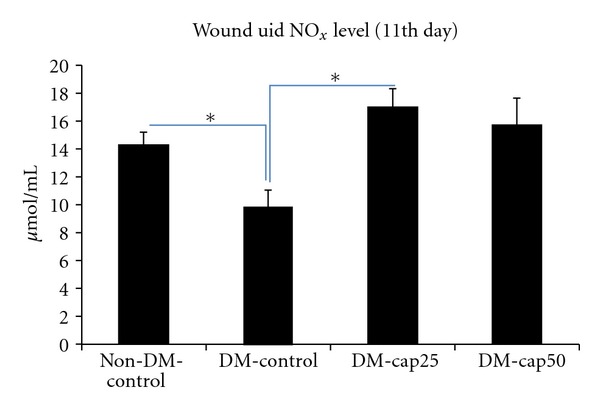
The total nitrite level of wound fluids was measured using Griess assay at the 11th day of study. Non-DM-control: nondiabetic rats which have received distilled water. DM-control: diabetic rats which have received distilled water. DM-cap25: diabetic rats which have received captopril (25 mg/kg/day). DM-cap50: diabetic rats which have received captopril (50 mg/kg/day). Data are reported in  *μ*mol/L ± SD (*P < 0.05).

At the 11th day, the wound fluid NO_*x*_ level of DM-cap25 group was more than other groups but the difference only between DM-cap25 group and DM-control group was significant (*P* < 0.002). The NO_*x*_ level of wound fluid in DM-cap50 group was significantly more than DM-control group (*P* < 0.007). Non-DM-control group had significantly more wound fluid NO_*x*_ level than DM-control group (*P* < 0.03) (Figure 3).

**Figure 4:**
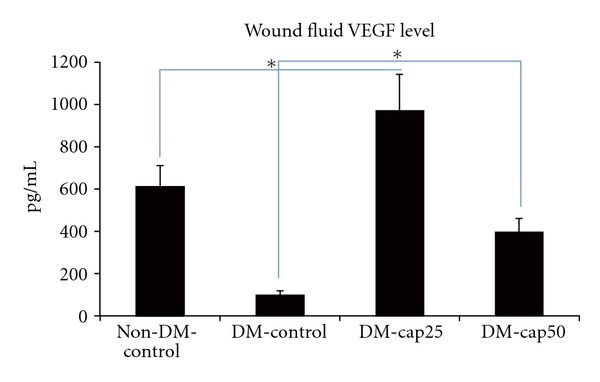
The VEGF level of wounds fluids was measured using ELISA at the 5th day of the study. Non-DM-control: nondiabetic rats which have received distilled water. DM-control: diabetic rats which have received distilled water. DM-cap25: diabetic rats which have received captopril (25 mg/kg/day). DM-cap50: diabetic rats which have received captopril (50 mg/kg/day). Data are reported in pg/mL ± SD (*P < 0.05).

The VEGF levels of wound fluid were only measured in the 5th day of the study. DM-cap25 group VEGF concentration was significantly higher than other groups (*P* < 0.02). VEGF concentration DM-cap50 group was significantly more than DM-control group (*P* < 0.05). Non-DM-control group had more VEGF concentration than DM-cap50 and DM-control groups, but the difference was significantly between non-DM-control and DM-control groups (*P* < 0.002) (Figure 4). 

**Figure 5:**
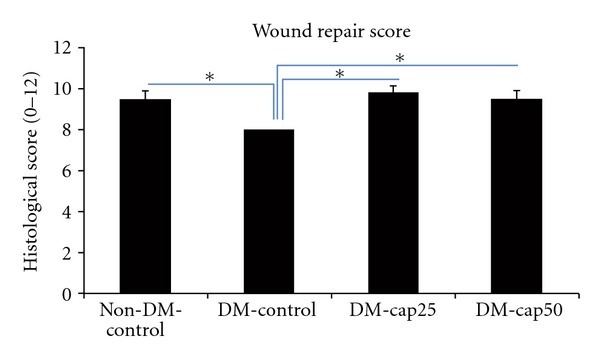
The histological scores of wound healing were examined by two pathologists, without knowledge of the prior treatment. Non-DM-control: nondiabetic rats which have received distilled water. DM-control: diabetic rats which have received distilled water. DM-cap25: diabetic rats which have received captopril (25 mg/kg/day). DM-cap50: diabetic rats which have received captopril (50 mg/kg/day). Data are reported in mean scores ± SD (*P < 0.05).

There was no significant difference in wound healing score between captopril-treated groups and non-DM-control group. The wound healing score in captopril-treated groups and non-DM-control group was significantly more than DM-control group (*P* < 0.05) (Figure 5). 

## 4. Discussion

The wound healing process involves a complex interplay of cells, mediators, growth factors, and cytokines [19]. The diabetic wound demonstrates a number of aberrations suggestive of localized endothelial dysfunction. Therefore, targeting of endothelial dysfunction may represent a promising focus for treatment of the diabetic wound. So, in this study, we tested whether captopril as a known effective drug on the endothelial dysfunction as well as frequently used drug in diabetic patients influences diabetic wound healing.

In our study, there was a significant decreased level of NO in wound fluid of diabetic rats. Decreased level of NO parallel to impaired healing has been shown in experimental diabetic animal models of wound healing [5]. Furthermore, abnormalities in endothelial and inducible NOS expression have been demonstrated in diabetic animal wound tissue when compared to nondiabetic counterparts [20, 21]. Captopril supplementation resulted in significantly increased levels of NO in wound fluid. ACEIs can increase NO production through increased available active kinins [22]. It has been shown that treatment of diabetic patients with ACEIs can improve NOS-dependent responses of large peripheral vessels [23, 24].

The significant different levels of wound fluid NO between the two doses of the ACE inhibitor in 5th day of the study may show a different dose-dependent effect of ACEIs on the NO.

It may be explained by an inverse relationship between the expression/activity of ACE and eNOS via feedback regulation [25]. It has been shown that the regulation of ACE is correlated with changes of the expression and activity of eNOS. ACE expression/activity is increased when eNOS expression/activity is decreased. Conversely, a decreased ACE expression/activity is observed when eNOS expression/activity is increased. Thus, it can be postulated that more ACE inhibition may be associated with more NO production. Furthermore, more ACE inhibition leads to further increase of endothelial-derived kinins and consequently more NO production. In agreement with our results, several other studies have reported dose-dependent effect of ACEIs on NO production [26].

VEGF concentration was significantly higher in both captopril-treated groups compared to both DM and non-DM control groups. It has been shown that impaired angiogenesis in diabetes is related to VEGF signaling and in inflammation-related pathway [27].

The results of several studies suggest that the effect of ACEIs on angiogenesis is related to ACEI-induced potentiation of endogenous bradykinin [28]. Li et al. showed that bradykinin receptor1 (BK-B1R) antagonist severely suppressed the imidapril-induced angiogenesis in angiotensin receptor 1a knockout (AT1aKO) mice, while NOS inhibitor moderately attenuated the imidapril-mediated angiogenesis [29]. Furthermore, it has been shown that proangiogenic effects of ACEIs are blunted in BK-B2R receptor-deficient diabetic mice [30].

Similar to our results, Gao and Yu have shown that ACEI treatment resulted in increased level of VEGF mRNA and protein expression by 1.44- and 1.30-fold compared with untreated diabetic rats in hind limb ischemic model [31]. Though, the combination of a BK-B1R antagonist to ACEI-treated group made the expression of VEGF decreased in their study.

In addition, NO can induce VEGF synthesis through an HIF-1-mediated pathway, and VEGF enhances NO production by eNOS [32, 33]. These actions may lead to promotion of angiogenesis. 

Furthermore, it has been shown that proangiogenic actions of Ang II could be mediated via the angiotensin type 1 (AT1) receptor, in part, through activation of VEGF-related pathway [34].

Several studies suggest that Ang II promotes cell proliferation and blood vessel growth [35, 36]. In line of these evidences, it can be speculated that low dose of ACEIs would be more effective in wound healing. It has been shown that very low dose of perindopril induces an early and sustained effect on the revascularization in ischemic tissue through a VEGF-dependent pathway [36].

In conclusion, it seems that low dose of captopril is safe and effective in diabetic wound healing. However, our study has some limitations. It would be better to study further doses of captopril and have a captopril-treated nondiabetic control group. Further studies especially well-designed clinical trials are required to confirm this finding.

## Figures and Tables

**Table 1:** Criteria to evaluate histological scores of wound healing.

Scores	
1–3	None to minimal cell accumulation, no granulation tissue or epithelial travel
4–6	Thin immature granulation, that is, dominated by inflammatory cells but has few fibroblasts, capillaries, or collagen deposition, minimal epithelial migration
7–9	Moderately thick granulation tissue can range from being dominated by inflammatory cells to more fibroblasts and collagen deposition, extensive neovascularization, epithelium can range from minimal to moderate migration
10–12	Thick, vascular granulation tissue dominated by fibroblasts and extensive collagen deposition, epithelium partially to completely covering the wound

**Table 2:** Mean levels of fasting plasma glucose in four study groups.

Group	DM-cap25	DM-cap50	DM-control	Non-DM-control
Fasting plasma glucose (mg/dL)	651 ± 89	526 ± 107	559 ± 59	119 ± 13*

*P < 0.05.
